# Sex and occupation time influence niche space of a recovering keystone predator

**DOI:** 10.1002/ece3.4953

**Published:** 2019-02-23

**Authors:** Erin U. Rechsteiner, Jane C. Watson, M. Tim Tinker, Linda M. Nichol, Matthew J. Morgan Henderson, Christie J. McMillan, Mike DeRoos, Marie C. Fournier, Anne K. Salomon, Leah D. Honka, Chris T. Darimont

**Affiliations:** ^1^ Department of Geography University of Victoria Victoria British Columbia Canada; ^2^ Hakai Institute Heriot Bay British Columbia Canada; ^3^ Vancouver Island University Nanaimo British Columbia Canada; ^4^ Department of Ecology and Evolutionary Biology University of California at Santa Cruz Santa Cruz California; ^5^ Nhydra Ecological Consulting St. Margaret's Bay Nova Scotia Canada; ^6^ Fisheries and Oceans Canada Pacific Biological Station Nanaimo British Columbia Canada; ^7^ Marine Education and Research Society Port McNeill British Columbia Canada; ^8^ School of Resource and Environmental Management Simon Fraser University Burnaby British Columbia Canada; ^9^ Salmon Watersheds Program Pacific Salmon Foundation Vancouver British Columbia Canada

**Keywords:** *Enhydra lutris*, foraging behaviour, intraspecific niche partitioning, niche space, predator effects, predator recovery, sea otter

## Abstract

Predators exert strong effects on ecological communities, particularly when they re‐occupy areas after decades of extirpation. Within species, such effects can vary over time and by sex and cascade across trophic levels. We used a space‐for‐time substitution to make foraging observations of sea otters (*Enhydra lutris*) across a gradient of reoccupation time (1–30 years), and nonmetric multidimensional scaling (nMDS) analysis to ask whether (a) sea otter niche space varies as a function of occupation time and (b) whether niche space varies by sex. We found that niche space varied among areas of different occupation times. Dietary niches at short occupation times were dominated by urchins (*Mesocentrotus* and *Strongylocentrotus *spp; >60% of diets) in open habitats at 10–40 m depths. At longer occupation times, niches were dominated by small clams (Veneroida; >30% diet), mussels (*Mytilus* spp; >20% diet), and crab (Decapoda; >10% diet) in shallow (<10 m) kelp habitats. Diet diversity was lowest (*H*′ = 1.46) but energy rich (~37 kcal/min) at the earliest occupied area and highest, but energy poor (*H*′ = 2.63, ~9 kcal/min) at the longest occupied area. A similar transition occurred through time at a recently occupied area. We found that niche space also differed between sexes, with bachelor males consuming large clams (>60%), and urchins (~25%) from deep waters (>40 m), and females and territorial males consuming smaller, varied prey from shallow waters (<10 m). Bachelor male diets were less diverse (*H*′ = 2.21) but more energy rich (~27 kcal/min) than territorial males (*H*′ = 2.54, ~13 kcal/min) and females (*H*′ = 2.74, ~11 kcal/min). Given recovering predators require adequate food and space, and the ecological interactions they elicit, we emphasize the importance of investigating niche space over the duration of recovery and considering sex‐based differences in these interactions.

## 1 INTRODUCTION

The effects of predators on ecological processes are determined by the suite of biotic and abiotic interactions that together comprise their niche space. The multidimensional niche space of a species (Hutchinson, 1957) can be examined by mapping a species’ biotic and abiotic requirements and then used to predict what interactions a species may have within a larger community (Holt, 2009). Recently, nutrition, physiology, and behaviour have also been used to better characterize niche breadth and predict a species’ trophic interactions (Machovsky‐Capuska et al., 2018). Although once viewed as static, niche space is no longer seen as a fixed property, but one that can change as a function of intraspecific variation (Baudrot, Perasso, Fritsch, Giraudoux, & Raoul, 2016; Ingram, Costa‐Pereira, & Araujo, 2018; Lafferty, Belant, & Phillips, 2015; Newsome et al., 2015). Factors such as life stage and habitat use (Polis, 1984), intraspecific competition (Newsome et al., 2015), and sex (Shine, 1989) all affect niche variation within species or populations. For example, niche space can change as a population reaches carrying capacity, reflecting increased intraspecific competition (Newsome et al., 2015). This has important implications for recovering populations of predators whose niche space may change over the course of recovery.

Globally, the extirpation of predators is known to have ecological consequences (Estes et al., 2011; Jackson, 2001), but an understanding of the roles of these predators in structuring ecological communities often occurs following reintroduction and subsequent recovery (i.e., Ripple & Beschta, 2012). When high trophic‐level species recover, they may have substantial effects on the trophically downgraded systems to which they return. As predators become reestablished, they may occupy new habitats, or broaden their diet, leading to increased diversity in niche space (i.e., Silliman et al., 2018). Recovering predator populations can also induce ecological changes that exert feedback on prey or habitat availability, further diversifying or expanding the predator's niche space (Estes, Jameson, & Rhode, 1982; Tinker, Bentall, & Estes, 2008).

Population diversification and expansion in niche space may reflect intraspecific differences in trophic interactions and habitat use brought about by sex‐specific constraints. Sex‐based differences in niche space have been demonstrated in marine vertebrates (Machovsky‐Capuska, Senior, Benn et al., 2016; Malinowski & Herzing, 2015) and may include body size differences or reproductive demands (Ruckstahl & Clutton‐Brock, 2005), or differing nutritional requirements (Machovsky‐Capuska, Senior, Simpson, & Raubenheimer, 2016). If the sexes occupy different niche space, their ecological needs and interactions might likewise vary. Thus, understanding how niche space is used by both sexes will provide a more comprehensive insight into the ecological effects and conservation needs of a species.

Sea otters (*Enhydra lutris*) provide a useful model to examine changes over time and sex‐related differences in niche space in a keystone predator recovering from near extinction. As a result of the Maritime Fur Trade that lasted from the mid‐1700s to the early 1900s, sea otters were likely ecologically extinct in British Columbia, Canada, by 1820–1850 (Sloan & Dick, 2012; Watson, 2000). Sea otters were reintroduced to Canada's west coast in the late 1960s and early 1970s (Bigg & MacAskie, 1978). Sea otters segregate by sex with nonterritorial males and adult females generally occupying discrete areas throughout the range (Riedman & Estes, 1990). As sea otter populations recover and recolonize habitats, groups of males pioneer range expansion, typically settling in urchin‐rich areas at the range edge (Lafferty & Tinker, 2014). Female dominated groups later replace male groups. This transition often occurs after the ecological community has changed from urchin‐ to kelp‐dominated as a result of sea otter foraging on sea urchins, an important reef herbivore (Lafferty & Tinker, 2014; Watson & Estes, 2011). As sea otter occupation time increases, kelp forest communities are further altered by the effects of otter foraging, and sea otter diets may become more diverse at the population level (Ostfeld, 1982; Tinker, Bentall et al., 2008).

Here, we use sea otters as a model to pose two broad questions about niche space within recovering carnivore populations. We made direct observations of sea otter foraging at five areas spanning a gradient of sea otter occupation time from 1 to 30 years to ask (a) how does the niche space of a predator change as the population recovers from extirpation and (b) how does niche space vary between females and males. Our findings yield insight into how ecological interactions exerted by predators vary during recovery and how these interactions can differ between female and male predators.

## 2 MATERIALS AND METHODS

### 2.1 Field methods

We observed sea otter foraging from 2014 to 2017 on the central coast of British Columbia in five *occupation areas,* where sea otters arrived at different periods: Gosling Rocks (1980s, occupied ~27–30 years), McMullin Islands (~1996, occupied ~18–21 years), Simond Islands (~2009, occupied ~5–8 years), Breadner Islands (~2011, occupied ~3–6 years), and Calvert Island (~2013, occupied 1–4 years). We estimated the arrival time of sea otters in an area based on the first reported observations of a raft of otters that occupied the area for a minimum of one year (Nichol, Watson, Abernethy, Rechsteiner, & Towers, 2015). We used a space‐for‐time design to estimate temporal changes in niche space (Pickett, 1989). At the most recently occupied area (Calvert), which was colonized by sea otters during our study, we divided longitudinal data into *Initial* (1 year occupied) and *Established* (2–4 years occupied) periods to examine potential changes in niche within an occupation area through time, thereby complementing the space‐for‐time design.

**Figure 1:**
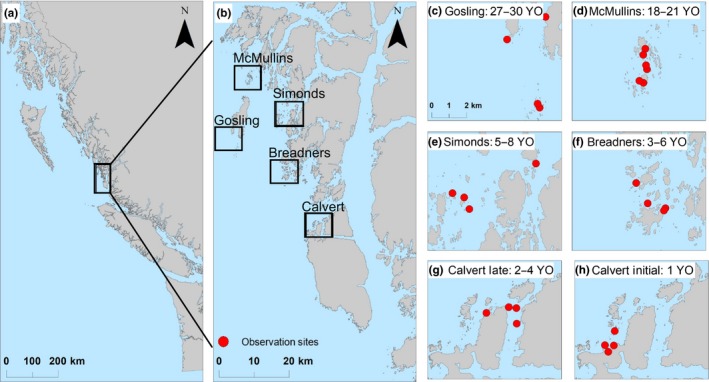
Map of British Columbia shoreline (a) and central coast study area (b) with occupation areas (c–h) and observation sites (red circles)

We collected data on diet and foraging behaviour using well‐established, standardized observation methods (Estes et al., 1982; Tinker, Bentall et al., 2008; Tinker et al., 2012). Data were collected in winter (January and February) and summer (June, July, August) by trained observers stationed at locations on shore within 1,000 m of foraging otters. Locations from which observers collected sea otter foraging data are called *observation sites* and were visited repeatedly throughout the four‐year study period (Figure 1).

We used a 50–80x magnification Questar telescope (Field model 3.5, Questar Corp., Pennsylvania, USA) to observe individual otters (identified by sex, age, and unique markings such as percent grizzled or blonde fur, animal size, and nose scarring patterns, which were unique for most individuals; i.e., Foott, 1970) over a contiguous sequence of dives, referred to as a foraging bout. For each dive, we recorded the duration of dive and surface intervals, dive outcome (i.e., whether prey were captured), prey type (identified to lowest taxon possible), and the number of items and size class of prey (relative to sea otter paw width; Supporting Information Figure S1; Estes et al., 1982; Tinker, Bentall et al., 2008; Tinker et al., 2012). We collected a minimum of 50 foraging bouts at each occupation area in each season and year, with the exception of the exposed Gosling Rocks site in winter (due to inclement weather), where we collected 60 bouts across all winters combined. Using species accumulation curves (Oksanen et al., 2018), we determined that a sample of 50 bouts accounted for ~95% of sea otter diet variability (Supporting Information Figure S2). Dean, Bodkin, and Coletti (2014) reported a similar sample size for sea otters in Alaska. We made observations within 1,000 m of large (>50–200 individuals) sea otter rafts; we worked at sites with many otters to increase the likelihood that 50 foraging bouts would include diverse individuals. We used individual markings to reduce the probability of recording multiple bouts in the same site and season by the same individual. Individual bouts were averaged to the observation site level, which was used as the replicate in all statistical models, avoiding the risk of pseudo‐replication.

### 2.2 Quantifying habitat use

We recorded several habitat parameters at each sea otter's surfacing location for each foraging dive. We used direct observation of floating canopy kelps (*Macrocystis pyrifera* or *Nereocystis leutkeana*) to determine the presence or absence of kelp where sea otters surfaced with prey. Seagrass (*Zostera* spp.) coverage was established through video tows made throughout the study area when underwater visibility was adequate (Hakai, 2014). Sea otter surface locations were spatially joined to seagrass polygons post hoc using ArcGIS (ESRI, 2017). The water depth foraged over by sea otters was determined post hoc using ArcGIS (ESRI, 2017) and was based on chart datum data obtained from the Canadian Hydrographic Service Bathymetry data (CHS 2016—license 2016‐03‐01‐1290‐H). Foraging depths were categorized as intertidal (<0 m below chart datum), shallow (0–10 m below chart datum), mid (10–40 m below chart datum), and deep (>40 m below chart datum).

### 2.3 Data analysis overview

Our analyses of foraging ecology and niche space variation in sea otters involved several steps. First, we used established analytical methods to quantify diet composition (i.e., frequency of occurrence representation of various prey taxa and size classes in sea otter diets), rate of energy gain, and diet diversity from observational foraging data (Hessing‐Lewis et al., 2017; Tinker, Bentall et al., 2008; Tinker et al., 2012). We then combined foraging data for each occupation area with environmental parameters (habitat, depth) and analyzed the multivariate data set using nonmetric multidimensional scaling analysis (nMDS) to examine whether there were groupings that correlated with occupation area or sex class. We used a similarity percentage analysis (SIMPER) to determine the diet items most strongly influencing similarities within groups and employed a hierarchical clustering analysis with group average linking to check for corroboration with nMDS results. Finally, we used an analysis of similarity (ANOSIM) test to determine whether or not differences among groups were significant. These analyses are described in detail below.

### 2.4 Foraging data analysis

We used direct observations of foraging behaviour combined with a Monte Carlo algorithm to analyze sea otter foraging data (Hessing‐Lewis et al., 2017; Tinker, Bentall et al., 2008; Tinker et al., 2012). Prey functional groups were defined by both taxa and size class (Table 1) and were limited to prey that comprised at least 5% of overall diets by frequency of occurrence. The proportion of each prey group and mean prey size from foraging bouts at the same observation site were averaged to provide site‐specific values for all prey groups. We then calculated the Shannon index of diversity (Shannon, 1948) for each occupation area. For the occupation area data, we limited consideration to observation sites with ≥25 bouts (*n* = 26 observation sites: four from Gosling, six from McMullins, four from Simonds, four from Breadners, four from Calvert *Established,* and four from Calvert *Initial*). For the sex class data, we further limited consideration to sites where ≥10 bouts were observed for at least one sex class. Sex classes were defined as *female *(*n* = 13 sites; all females regardless of reproductive status), *territorial male* (*n* = 11 sites; males holding territories), or *bachelor male* (*n* = 12 sites; males aggregated in large male‐only rafts, which in our study existed both in the range centre and at the range edge). In California (Jameson, 1989; Tarjan, 2016; Tinker, Doak, Doak, & Estes, 2008) and Alaska (Garshelis & Garshelis, 1984), some males move between bachelor groups and female areas where they may become territorial males seasonally, and we suspect this is also the case in British Columbia. Therefore, classifications of *territorial* or *bachelor* male in our study apply to the otter at the time and location it was observed foraging.

**Table 1:** Prey groups used in nonmetric multidimensional scaling analysis and cluster analyses

Common name	Lowest taxon	Size (paw)	Apx. size (cm)	Prey group	Prey group abbreviation
Urchin	*Mesocentrotus* spp.*, Strongylocentrotus *spp.	1a−1c	<2–5	Small urchin	urc_sm
		2a−4c	7–20	Large urchin	urc_lrg
Clam	Veneroida	1a−1c	<2–5	Small clam	clam_sm
		2a−4c	7–20	Large clam	clam_lrg
Chiton	Polyplacophora	1a−1c	<2–5	Small chiton	chit_sm
		2a−2c	7–10	Medium chiton	chit_med
		2a−6c	7–30	Large chiton	chit_lrg
Abalone	*Haliotis kamtschatkana*	1a−1c	<2–5	Small abalone	aba_sm
		2a−3c	7–15	Large abalone	aba_lrg
Cucumber	Holothuroidea	1a−1c	<2–5	Small cucumber	cuc_sm
		2a−2c	7–10	Medium cucumber	cuc_med
		2a−8c	7–40	Large cucumber	cuc_lrg
Crab	unknown crabs	1a−1c	<2–5	Small crab	crab_sm
		2a−4c	7–20	Large crab	crab_lrg
Cancer crab	*Cancer* spp.	1a−1c	<2–5	Small cancer crab	can_sm
		2a−4c	7–20	Large cancer crab	can_lrg
Geoduck	*Panopea generosa*	1a−2c	<2–10	Small geoduck	geo_sm
		3a−3c	12–15	Medium geoduck	geo_med
		4a−4c	17–20	Large geoduck	geo_lrg
Kelp crab	*Pugettia *spp.	1a−1c	<2–5	Small kelp crab	kelpcrab_sm
		2a−3a	7–12	Large kelp crab	kelpcrab_lrg
Mussels	*Mytilus* spp.	1a−1c	<2–5	Small mussel	mus_sm
		2a−4c	7–20	Large mussel	mus_lrg
Scallop	Crassadoma	1a−1c	<2–5	Small scallop	scal_sm
		1c−4a	5–17	Large scallop	scal_lrg
Snail	Turbinidae	1a−1c	<2–5	Small snail	sna_sm
		2a−3c	7–15	Large snail	sna_lrg
Octopus	Octopoda	All	All	Octopus	oct
Shore crab	*Hemigrapsus *spp.	All	All	Shore crab	shorecrab

Energy intake rates can be used to infer the abundance and quality of prey resources for sea otter populations (Tinker, 2015). Because population growth in sea otters is usually determined by prey abundance, energy intake provides a useful index of status with respect to carrying capacity (i.e., Tinker, Bentall et al., 2008; Tinker et al., 2012). Data on size‐specific edible biomass and caloric content of most sea otter prey items are available from an earlier study (Oftedal, Ralls, Tinker, & Green, 2007). These data can be combined with observational foraging data to estimate energy intake rates for sea otters. Sea otters are almost unique in their tractability for foraging studies, because their feeding dives are conducted close to shore and prey are consumed at the surface, where prey species, size, and handling time can be readily observed. However, recording all relevant parameters is subject to the challenges of direct observation, and missing information tends to be biased toward smaller prey, shorter handling times, or more distant sea otters (Tinker, 2015; Tinker et al., 2012). To account for this potential bias, we used a Monte Carlo algorithm used in many previous studies (e.g., Tinker et al., 2012; Newsome et al., 2015; Hessing‐Lewis et al., 2017). This process‐based model replicates the recorded foraging bouts and iteratively assigns missing parameters by drawing randomly from appropriate probability distributions while maintaining observed covariance patterns between dive parameters (details in Tinker et al., 2012; Tinker, 2015). The results of the analysis include estimates of energy intake rates (kcal consumed per minute of time spent feeding) for sea otters at each observation site.

### 2.5 Niche space analysis

We quantified niche space along multiple dimensions corresponding to prey selection and habitat and depth‐use variables. As with the foraging data, variables were first quantified at the bout level and then averaged at the site level with each site average becoming an individual point in the nMDS analysis. Variables used in the nMDS analysis included the proportional representation of prey functional groups, dive habitat (open, kelp canopy, seagrass), and depth (intertidal, shallow, mid, deep) over which sea otters foraged.

We used PRIMER‐e V7 (Clarke & Gorley, 2015) to calculate a resemblance matrix of sea otter niche space at each observation site and for each site‐sex class pair, using Bray–Curtis dissimilarities of square‐root‐transformed diet, habitat, and depth variables (Clarke, Gorley, Somerfield, & Warwick, 2014). To include the three different data types (diet, habitat, and depth) in the resemblance matrices, we square‐root‐transformed all data and then rescaled the habitat and depth variables such that the mean values across sites were equal to the mean of the transformed prey values; this procedure prevented any single data type (diet, habitat, or depth) from having an exaggerated influence on the nMDS analysis (Clarke et al., 2014; Kenner & Tinker, 2018).

The resemblance matrices were then used to perform nonmetric multi dimensional scaling (nMDS) in PRIMER‐e (Clarke & Gorley, 2015). We incorporated environmental data (depth and habitat), mean prey size, diet diversity, energy intake rate, and occupation time as vectors in our nMDS plots post hoc. Correlations between each of these vectors and the ordination axes were calculated with the Pearson correlation coefficient (Clarke et al., 2014). Vectors with correlation coefficients ≥0.50 were included on the nMDS plots. The nMDS converged in two dimensions with stress values of 0.12 (occupation areas) and 0.13 (sex). An nMDS with stress values below 0.20 can provide a useful two‐dimensional interpretation, but if stress is >0.10, the plot should be superimposed with results from cluster analyses to assess agreement (Clarke et al., 2014). Thus, for each resemblance matrix, we also performed hierarchical clustering using group average linking of replicate sites at each occupation area or for each sex (Clarke et al., 2014).

We used an ANOSIM test to determine whether groups (occupation areas, sex classes) were significantly different (Clarke et al., 2014). If significant differences were detected in the global ANOSIM test, pairwise comparisons were made. We used a SIMPER analysis to determine which prey and environmental metrics contributed most to similarities within clusters identified by nMDS and included the most influential prey groups in bubble plots to help interpret nMDS results (Clarke et al., 2014).

## 3 RESULTS

### 3.1 Sea otter foraging data

We observed 19,535 foraging dives in 1,983 bouts. Of these, 12,922 foraging dives from 1,330 bouts were collected from observation sites where we had observed ≥25 bouts. Individual bouts ranged from 3 to 81 dives (mean 10.10 ± 0.22 dives *SE*) and from 2.4 to 162 min (mean 20.8 ± 0.48 min *SE*). We identified the sex of the otter in 968 (~73%) of these bouts, which were used for the nMDS analysis with sex. Prey sizes and energy intake are reported as mean ± *SE*.

**Figure 2:**
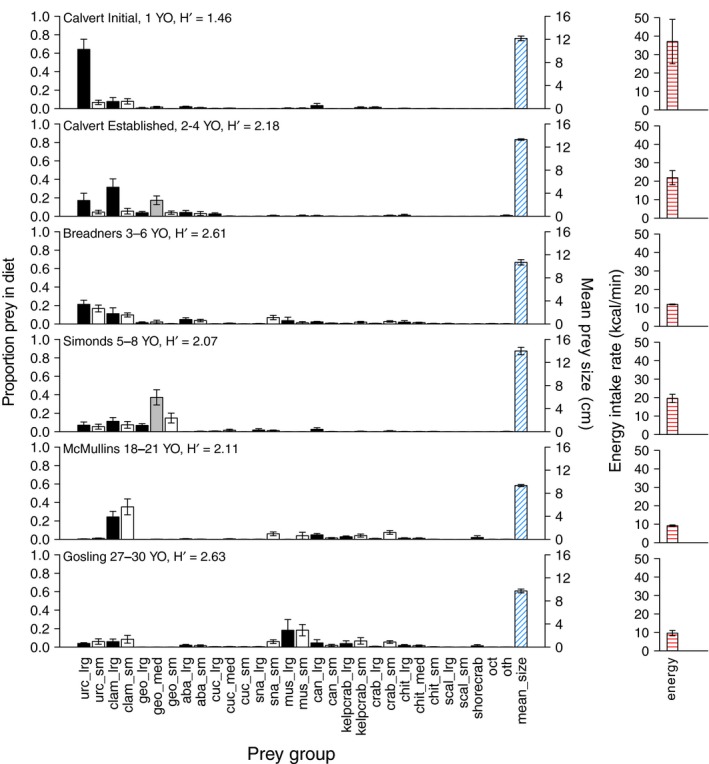
Prey consumed by sea otters at occupation areas from 1 to 30 years occupied (YO) as proportion of diet by frequency of occurrence. Black bars show large prey, gray bars show medium prey, and white bars show small prey. Blue cross‐hatched bars indicate mean size of prey. Red cross‐hatched bars show energy intake. Error bars are *SEM*, *n* = 4 for all occupation areas except McMullins where *n* = 6. *H*′ is Shannon index of diversity. For prey group abbreviations, see Table 1

Diet varied among sites with different occupation times. Sea otters at the Calvert *Initial* area consumed ~60% large urchins (*Mesocentrotus* and *Strongylocentrotus *spp.) while large urchins composed only ~20% of otter diets at Calvert *Established* and Breadners (3–6 years occupied), ~10% of diets at Simonds (5–8 years occupied), and less than 2% of diets at the McMullins (18–21 years) and Gosling (27–30 years) (Figure 2). The proportion of clams (Veneroida), including geoduck (*Panope generosa*), comprised <25% of the diet at Calvert *Initial* and Breadners but was>50% of the diet at Calvert *Established*, Simonds, and McMullins (Figure 2). Sea otter diets at Gosling Rocks were composed mostly of mussels (*Mytilus* spp. ~40%), clams (~10%), urchins (~10%), and kelp crabs (*Pugettia* spp. ~10%) (Figure 2). Mean prey size declined from 12.17 ± 0.41 cm at Calvert *Initial* to 9.73 ± 0.33 cm at Gosling Rocks (Figure 2). Shannon indices showed lowest diversity (*H*′ = 1.46) at Calvert *Initial* and higher diversity (*H*′ = 2.07–2.63) at all other occupation areas (Figure 2). Monte Carlo analysis indicated that the mean energy intake rate declined from 37.04 ± 12.02 kcal/min consumed at Calvert *Initial* to 9.61 ± 1.45 kcal/min at Gosling Rocks (Figure 2).

**Figure 3:**
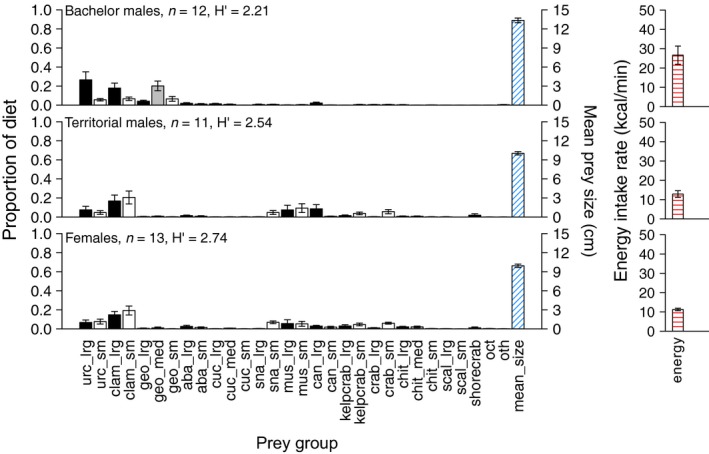
Prey consumed by sea otters of each sex class as proportion of diet by frequency of occurrence. Black bars show large prey, gray bars show medium prey, and white bars show small prey. Blue cross‐hatched bars indicate mean size of prey. Red cross‐hatched bars show energy intake. Error bars are *SEM*, n is number of sites, and *H*′ is Shannon index of diversity. For prey group abbreviations, see Table 1

Sea otter diets also differed by sex. Bachelor males consumed ~30% urchins, and >50% clams, whereas territorial males and females consumed ~15% urchins, ~20% each of small and large clams, >10% each of small crabs and mussels, and ~10% snails (Turbinidae; Figure 3). Mean prey size was highest for bachelor males (13.34 ± 0.37 cm) and lower for territorial males (10.03 ± 0.26 cm) and females (9.94 ± 0.26 cm) (Figure 3). Shannon indices showed lowest diversity in bachelor male diets (*H*′ = 2.21) and higher diversity in territorial male and female diets (*H*′ > 2.50; Figure 3). Monte Carlo analysis indicated that the mean energy intake rate was highest for bachelor males (26.67 ± 4.72 kcal/min) and lower for territorial males (12.89 ± 1.72 kcal/min) and females (11.29 ± 0.63 kcal/min; Figure 3).

### 3.2 Sea otter niche space

**Figure 4:**
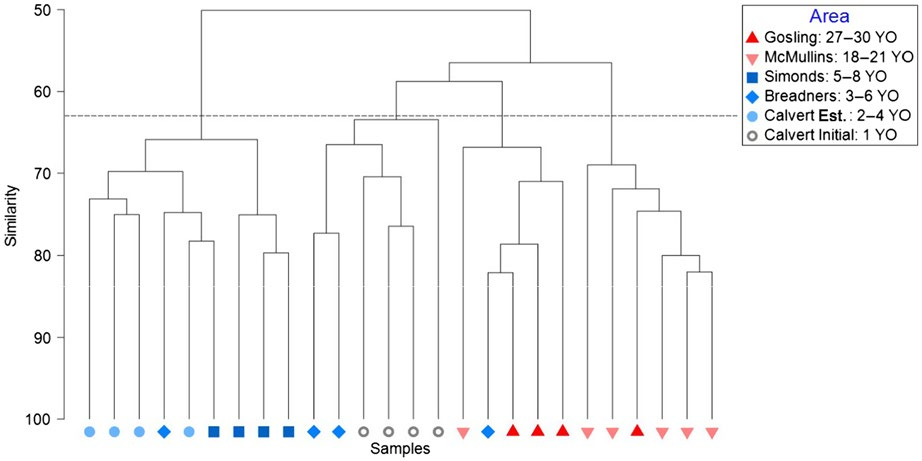
Dendrogram of hierarchical clustering (using group average linking) of replicate observation sites at each occupation area, based on Bray–Curtis dissimilarity matrix of sea otter diets. Dotted line shows 63% similarity. Grey symbols correspond to the shortest occupation time, blue symbols to medium occupation times, and red symbols to longest occupation times

**Figure 5:**
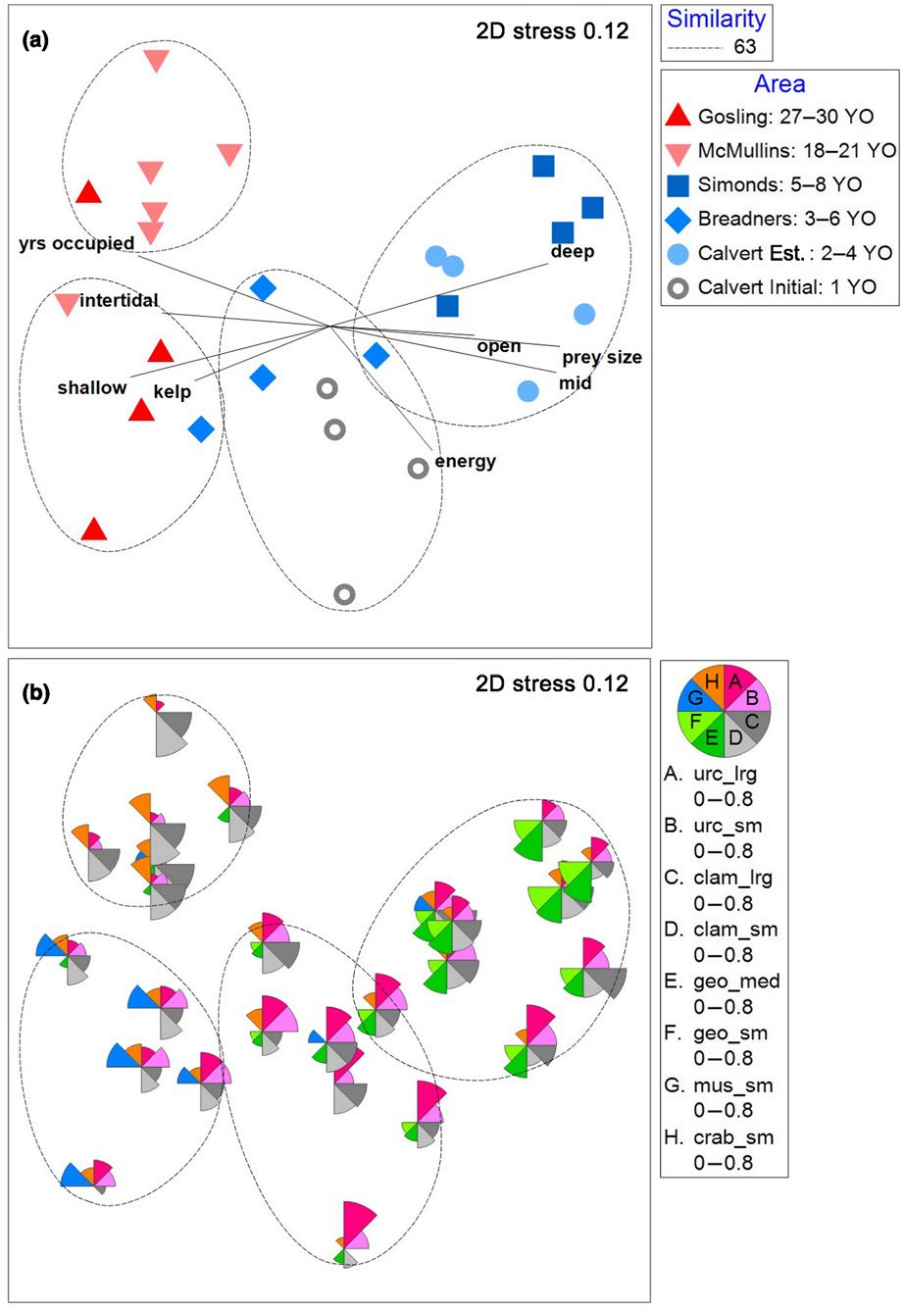
Nonmetric multidimensional scaling analysis plot of sea otter niche space with (a) clusters identified in Figure 4 with 63% similarity, and environmental vectors with ≥0.5 correlation to dissimilarities, and (b) bubble plots depicting the most common prey groups, with bubble segments approaching sizes of segments in the legend representing ~80% of the diet by frequency of occurrence

Otters from different occupation areas strongly diverged in niche space. Cluster analysis showed groupings with 63% similarity by occupation area and by *Initial* and *Established* sites at the Calvert occupation area (Figure 4). Results of the nMDS showed two‐dimensional stress of 0.12 and dissimilarities among occupation areas (Figure 5a). The results of the ANOSIM supported statistically significant differences in niche space among occupation areas (ANOSIM, *R* = 0.74, *p* < 0.001). All but two pairwise comparisons were significantly different from one another (Supporting Information Table S1). Bubble plots depict the prey species most important in driving similarities within occupation areas, as identified by SIMPER (>8% contribution to within‐group similarity; Figure 5b). We found that niche space similarities within occupation areas were determined primarily by urchins, clams, geoduck, mussels, small crabs, open water (i.e., absence of kelp canopy), and shallow water (Supporting Information Table S2). Post hoc vector correlations indicated that use of intertidal, shallow, and kelp canopy areas was correlated with longer occupation times, whereas use of deep, mid, and open areas, large prey sizes, and higher rates of energy intake were correlated with shorter occupation times (Figure 5a, Supporting Information Table S3).

**Figure 6:**
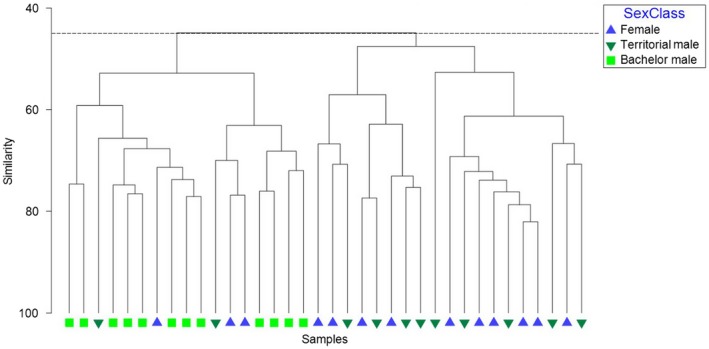
Dendrogram of hierarchical clustering (using group average linking) of replicate observation sites for each sex, based on Bray–Curtis dissimilarity matrix of sea otter diets. Dotted line shows 45% similarity

**Figure 7:**
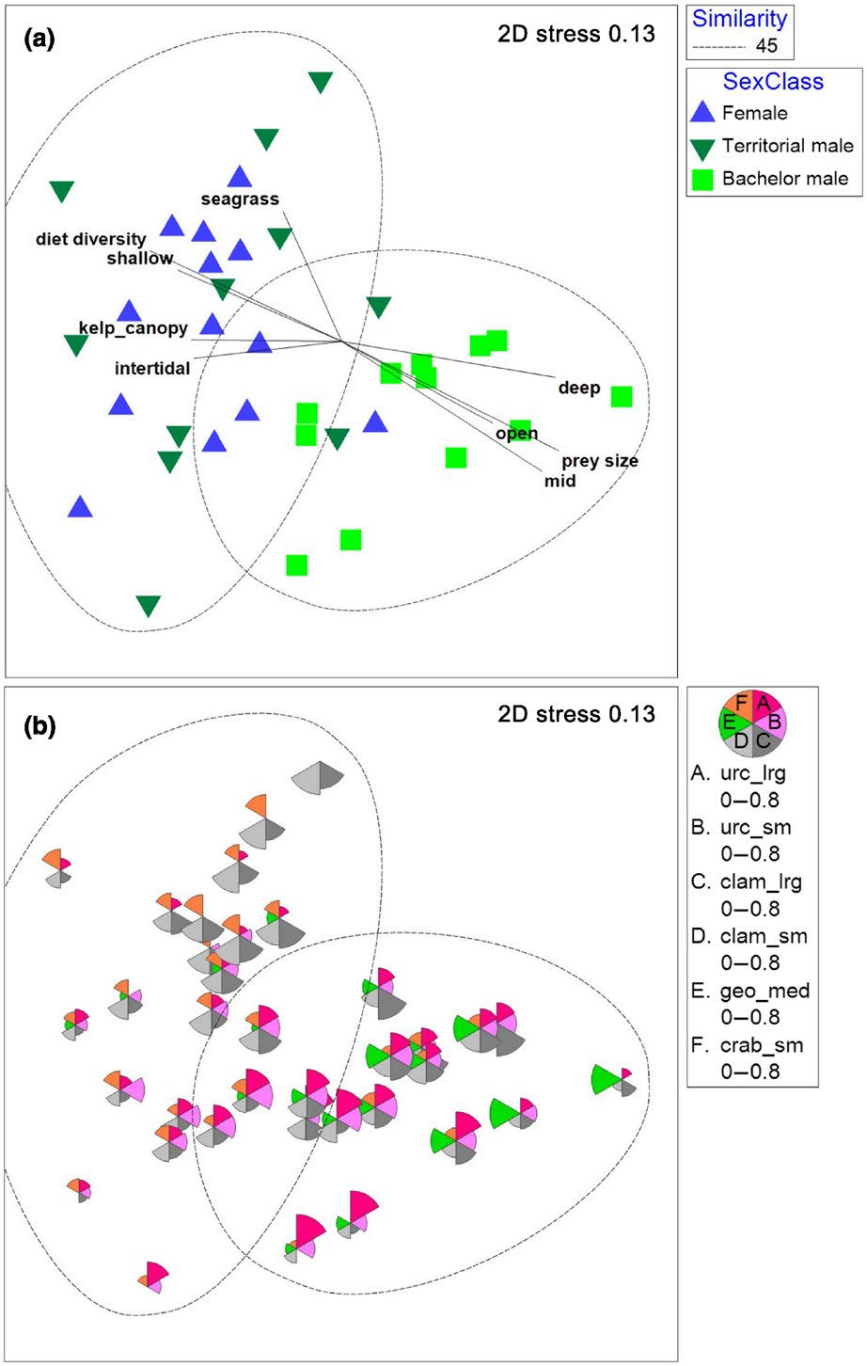
Nonmetric multidimensional scaling analysis (nMDS) plot of sea otter niche space with (a) clusters identified in Figure 6 with 45% similarity, and environmental vectors with ≥0.5 correlation to dissimilarities, and (b) bubble plots depicting the most common prey groups, with bubble segments approaching sizes of segments in the legend representing ~80% of the diet by frequency of occurrence

Sexes also showed strong divergence in niche space. Cluster analysis identified groupings by sex class with 45% similarities (Figure 6). Results of the nMDS showed two‐dimensional stress of 0.13 and dissimilarities among females and territorial males, and bachelor males (Figure 7a). The results of the ANOSIM confirmed that niche space significantly differs among sex classes (ANOSIM, *R* = 0.36, *p* < 0.001). Pairwise comparisons revealed that females and territorial males used a different niche space than bachelor males (Supporting Information Table S4). Bubble plots depicting the variables most important in driving similarities within sex classes, as identified by SIMPER (>8% contribution to within‐group similarity; Figure 7b), illustrate differences in niche space were driven by urchins, clams, geoduck, small crabs, open water, and shallow water (Supporting Information Table S5). Post hoc vector correlations indicated that higher diet diversity and use of intertidal, shallow, kelp canopy, and seagrass areas were correlated with females and territorial males, whereas use of deep, mid, and open areas, and larger prey sizes were correlated with bachelor males (Figure 7a, Supporting Information Table S6).

## 4 DISCUSSION

### 4.1 The dynamics of sea otter niche space

In this study, we used sea otters as a model to evaluate support for the hypotheses that a) predator niche space changes during population recovery and b) niche space varies between sexes. We found that across a gradient of occupation times, sea otters on the BC central coast occupied different niche spaces, initially foraging on large urchins in open water, before transitioning to soft‐sediment habitats to forage on clams in deep waters, and then to kelp canopy and intertidal areas where smaller and more diverse invertebrates were acquired. Both mean prey size and energy intake rates were lower in long‐occupied areas than in recently occupied areas. These niche space differences among sites with varying occupation times likely reflect sea otter behavioural responses to variation in prey availability—a legacy of the effects that sea otters exert on benthic prey communities as they recover (i.e., Estes, Riedman, Staedler, Tinker, & Lyon, 2003; Tinker, Bentall et al., 2008). The pattern we observed at a single occupation area over the four‐year study period offers evidence to support this hypothesis, with sea otter diets changing from urchin‐dominated (>60%) to clam‐dominated (~ 50%) after only ~1 year of occupation.

Niche space also varied among female, territorial male, and bachelor male sea otters. Bachelor males at recent and more established areas (i.e., 1–8 years) foraged predominantly in open (no kelp canopy or seagrass) habitats, using deeper waters than females, and consumed ~70% clams. In contrast, females and territorial males exhibited higher diet diversity and used kelp canopy, seagrass, and shallow and intertidal waters to forage on a diverse suite of smaller prey. Together, these findings suggest that the niche occupied by sea otters on the central coast of BC is context‐specific, depending on both occupation time and sex.

Although niche space has not been thoroughly examined in sea otters (but see Silliman et al., 2018), sea otter diets have been well studied. In British Columbia (Breen, Carson, Foster, & Stewart, 1982; Hessing‐Lewis et al., 2017; Honka, 2014), Washington (Laidre & Jameson, 2006; Hale et al., in press), California (Estes et al., 1982, 2003; Tinker, Doak et al., 2008; Tinker et al., 2012), and Alaska (Hoyt, 2015; Kvitek, Oliver, DeGange, & Anderson, 1992; Weizman, 2013), sea otter diets have been found to differ among recently and long‐occupied areas. In California, where individual sea otters have been observed for decades, sea otter diets diversify at the population level as occupation time increases (Estes et al., 1982). In Washington State, sea otter diets are affected more by kelp habitat than occupation time and population density (Hale et al., in press). On the exposed Washington coast, kelp habitats may be an important source of food, as well as providing shelter from storms and predators (i.e., Thometz et al., 2016), whereas in BC, the role of kelp may be less important because there is more shelter provided by islands and inlets (Hessing‐Lewis et al., 2017).

Differences in the diets of female and male sea otters have also been noted in other regions. In California, individual males over 1.5 years consumed more diverse prey than females, though this likely included males in transition between bachelor and territorial stages (Elliot‐Smith, Newsome, Estes, & Tinker, 2015). In our study bachelor males had the least diverse diets, with large groups of bachelor males (n = ~50‐150) often moving en masse (within ~1.5 km) to new rafting and foraging areas where prey were large and foraging success was high. In contrast, females may restrict their diets to increase time spent at the surface with their pups (Thometz et al., 2016). These sex‐based niche differences are important particularly because the feeding ecology and habitat use of large male groups, which occur across the geographic range of sea otters, have been largely overlooked.

### 4.2 Study limitations

Our study indicates that changes occur in sea otter niche space with increasing occupation time, changes that are likely a product of the ecological effects exerted by the sea otters themselves. However, the inherent weakness of space‐for‐time frameworks lies in determining the extent to which differences are a function of predator recovery and occupation time, or reflect variation among sites in the absence of predator recovery. Our observational design mitigates this limitation. We used multiple observation sites (*n* = 4–6) within each occupation area to replicate occupation time and monitored changes within each occupation area over the four‐year study period. Only Calvert, the most recently occupied area, showed any indication of niche change, and we suspect this is because initial changes happen rapidly, whereas further changes take longer than 4 years to observe. To interpret whether, for example, sea otter diets at longer occupation times transition from clam‐dominated to mussel‐dominated and smaller invertebrate‐dominated (as appears to be the case when comparing McMullins to Gosling), we would need to observe otters and the associated prey communities in each occupation area on a timescale of decades. This would also confirm whether the changes detected from our space‐for‐time design are indeed a factor of the ecological effects of sea otters on the prey community (e.g., Tinker, Doak et al., 2008; Tinker et al., 2012).

Our study focused on the energy intake, diet, and habitat use of sea otters. Sea otters, lacking the insulating blubber of most marine mammals, have high metabolic rates and consume ~25% of their bodyweight daily (Costa & Kooyman, 1982). Previous research suggests that most critical macro‐ and micronutrients are well represented in sea otter diets and that energy is the primary resource driving sea otter prey selection (Oftedal et al., 2007). However, variability in macronutrient composition among prey species can be an important driver of diet choice and niche space (Machovsky‐Capuska et al., 2018; Machovsky‐Capuska, Senior, Simpson et al., 2016; Mayntz, Raubenheimer, Salomon, Toft, & Simpson, 2005; Raubenheimer, Simpson, & Mayntz, 2009; Tait, Raubenheimer, Stockin, Merriman, & Machovsky‐Capuska, 2014). Accordingly, including data on the macronutrient content of prey may enhance our characterization of niche space, but quantifying macronutrient profiles for the diverse suite of prey that sea otters consumed was beyond the scope of this study. However, future work should include a more comprehensive examination of nutritional dimensions to sea otter prey selection.

Energy intake was approximated by an established analytical framework used in many sea otter foraging studies (e.g., Tinker, Bentall et al., 2008; Tinker et al., 2012; Hessing‐Lewis et al., 2017). This analysis makes use of a published data set on prey edible biomass and caloric content from 76 taxa collected in all seasons over a 4‐year period at multiple sites in California and Alaska (see Oftedal et al., 2007; Tinker, 2015 for more details). Although these data are broadly representative, we recognize their limitation for assessing fine‐scale variation in prey quality. While such fine‐scale variation is undoubtedly important, our study was designed to examine coarse‐scale patterns of niche use. Because we collected data over a 4‐year period and sampled equally across winter and summer, we believe that we captured variation in sea otter diets and energy intake at the scale of interest.

Disentangling the relative effects of sex and occupation time in sea otter niche space is difficult because the two are linked via the natural history of sea otters. On the central coast of BC, groups of bachelor males (typically *n *= >100 males in each raft) occurred at the range edge (Calvert *Initial* and *Established*) and in the range centre (Simonds). Unpublished data (50 foraging bouts) collected over a two‐week period in August 2016 by EUR and JCW in Kyuquot Sound (50.0°N, 127.4°W; >200 km south of the central coast of BC), in an area occupied by bachelor male sea otters since the 1980s, corroborated our findings: This bachelor male group foraged primarily on small clams (~70% of diet) in open waters. Further unpublished data (50 foraging bouts) collected over a two‐week period in July 2017 by LMN and CJM in the Nuchatlitz Islands (49.8°N, 126.9°W; ~40 km south of Kyuquot Sound), at an area occupied by sea otters since the 1990s, similarly found that the diet of bachelor males was comprised mostly of clams (~70%). Our findings indicate that even at long‐occupation times, bachelor males exploit a soft‐sediment, open‐water niche space where they feed on clams; however, at longer occupation times, clams were small and energy intake rates were likely lower than on the central coast of BC.

### 4.3 Ecological and conservation implications of changing niche space in sea otters

Sex‐related differences in feeding ecology and habitat use are rarely considered in habitat management or ecological interactions, despite these differences having potentially major effects (Du Toit, 2005). In a polygynous species such as the sea otter, the niche used by males—and the associated effects on survival—may be less important to population productivity than the niche used by females, because individual females contribute more to population recovery than males (Emlen & Oring, 1977; Tarjan, 2016). Our finding that females gain less energy per minute of foraging than bachelor males indicates that they must spend more time foraging than males. Moreover, the high cost of lactation (Thometz et al., 2016) and parental care may put females at a greater conservation risk than males. This finding suggests that site fidelity in females, even to lower‐energy prey areas, is important, perhaps to avoid predators or due to other reproductive constraints. Thus, recovery planning should consider that females and territorial males may require different prey and habitat than bachelor males.

Understanding how prey selection and the range of habitats used by sea otters vary over the course of recovery has important implications for sea otter conservation and affects our understanding of the recovery process in a predator that has traditionally been studied in a limited context (Silliman et al., 2018). Although dietary changes have been noted within and among sea otter populations, only limited research has examined niche variation across space and time. Following the Maritime Fur Trade, most remnant populations of sea otters occurred along the outer coast of the Pacific Rim. Thus, much of the foundational work on sea otter ecology comes from a limited ecological context—that of otters in open, exposed rocky substrate and kelp forest habitats (i.e., Estes & Palmisano, 1974; Estes & Duggins, 1995). Although research has focused on the role of sea otters in hard substrate areas, they use soft substrate areas as well, at both recent‐ and long‐occupied sites (Kvitek, Fukayama, Anderson, & Grimm, 1988; Kvitek et al., 1992; Weizman, 2013) and particularly in stormy weather (Garshelis & Garshelis, 1984). Only recently have the effects of sea otters in estuaries and salt marshes been examined (Hessing‐Lewis et al., 2017; Hughes et al., 2013, 2016), and such habitats are beginning to be considered essential, and historically important, to sea otters (Silliman et al., 2018). In our study, we found that use of soft‐sediment habitat becomes more important over the course of recovery and that soft‐sediment communities may be more important to bachelor males than females.

Although the ecological effects of sea otters foraging on urchins at exposed rocky sites are well known (see Estes, Heithaus, McCauley, Rasher, & Worm, 2016 for a review), their effects on other habitat types and prey communities are less well understood. Sea otter interactions in contexts other than urchin–kelp communities include serial depletion of macroinvertebrate prey (Salomon, Tanape, & Huntington, 2007), homogenization of mussel beds (Singh et al., 2013), and clearing patches in mussel beds that provides space for diverse invertebrate recruits (VanBlaricom, 1988), which can in turn affect shorebird diets (Berg, 2015). In soft‐sediment areas, sea otters can reduce large size classes of clams and alter benthic community structure as their occupation time increases (Kvitek et al., 1992; Weizman, 2013). Sea otter predation on clams may have further effects in seagrass communities: Digging could act as a source of disturbance to plants (i.e., Alexandre, Santos, & Serrão, 2005) and invertebrates (Kvitek et al., 1992). Recently, the role of sea otter predation on crabs in seagrass communities has been shown to initiate a trophic cascade that increases seagrass biomass (Hughes et al., 2013). These studies show that sea otter community interactions are context‐dependent and that the ecological consequences of alternative niche‐use patterns may be important to consider in terms of beneficial conservation impacts, fisheries interactions, and other management and conservation priorities (Silliman et al., 2018).

### 4.4 The dynamics of niche space in recovering predators

Most of the world's large predators have suffered widespread extirpation, limiting our understanding of how ecological communities function with intact predator populations (Estes et al., 2011; Jackson, 2001; Silliman et al., 2018). Thus, the interactions among predators, herbivores, and primary producers, and the habitat and food requirements of recovering predators, are difficult to predict. Over the course of recovery, many predators have expanded their ecological niche, often surprising ecologists with the different ecological interactions that they can elicit (Silliman et al., 2018).

Niche partitioning, once seen as a static species characteristic (Hutchinson, 1957), is now viewed as a dynamic property driven by intraspecific differences in traits such as age and sex (Polis, 1984; Shine, 1989), as well as factors such as population size (Newsome et al., 2015), and historical context (Jackson, 2001; Silliman et al., 2018). We used a reintroduced sea otter population to examine these ideas and found that niche space differed between sexes and changed over time as sea otter recovery progressed. These results have implications for depleted or recovering predator populations because sex‐related differences and temporal changes in characteristics such as feeding ecology and habitat use will affect the recovery process. If ecologists treat niche space as being dynamic and intraspecifically partitioned, they will be better equipped to predict the conservation needs and ecological interactions of recovering predators and to consider more broadly the ecological interactions that may have been driven by predators historically.

## CONFLICT OF INTEREST

None declared.

## AUTHORS’ CONTRIBUTIONS

EUR, JCW, MTT, LMN, AKS, LDH, and CTD conceived the ideas and designed the methodology. EUR, JCW, MMH, CJM, MDR, MCF, and LMN collected the data. EUR and MTT analyzed the data. EUR led the writing of the manuscript. All authors contributed critically to the drafts and gave final approval for publication.

## Data Availability

Data are archived on the Dryad Digital Repository, DOI: https://doi.org/10.5061/dryad.1v3g322.
