# Supplementary figures and images for: Sex and occupation time influence niche space of a recovering keystone predator
Source: Ecol Evol. 2019 Feb 23;9(6):3321–34. doi: 10.1002/ece3.4953 (PMC6434543; doi:10.1002/ece3.4953)

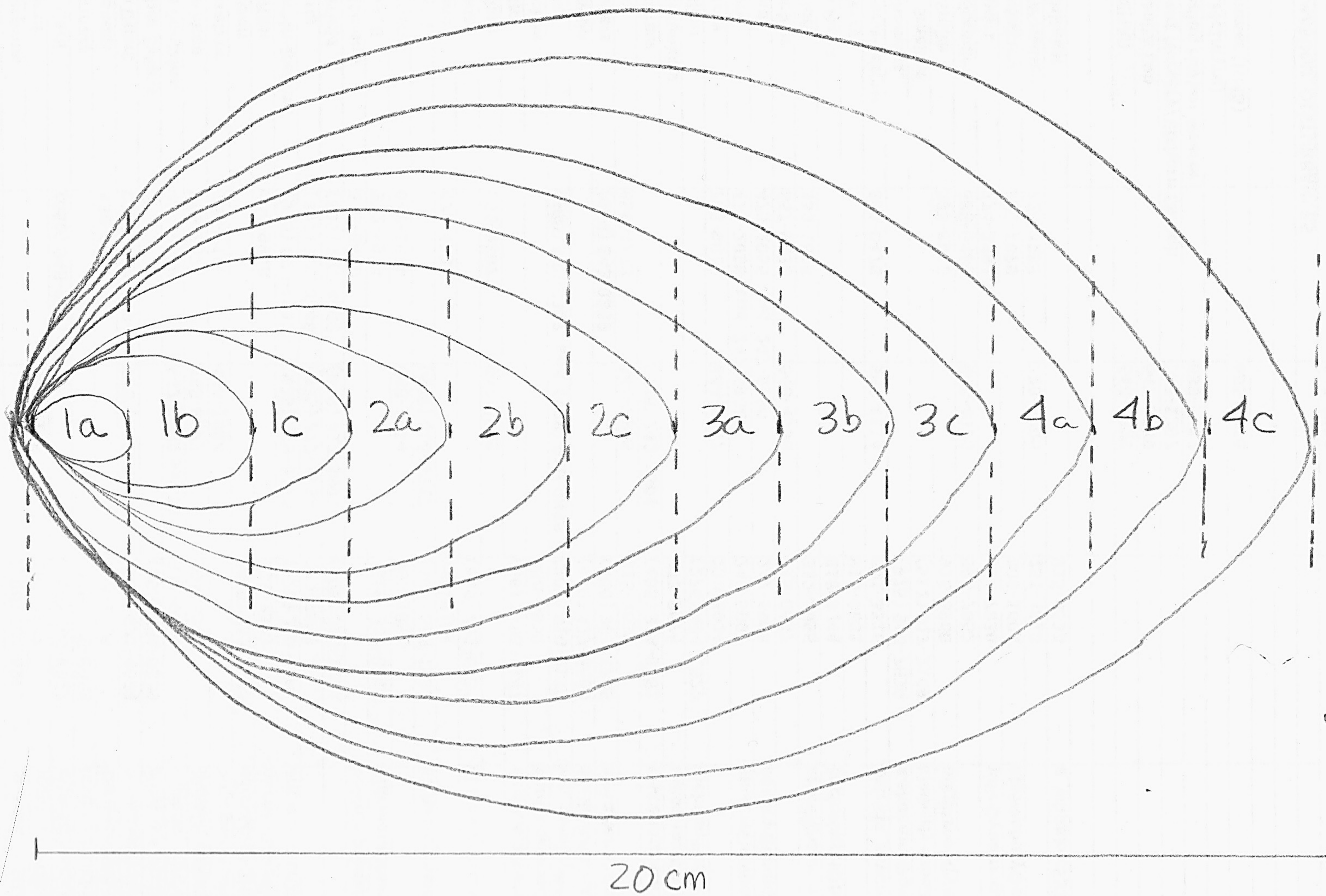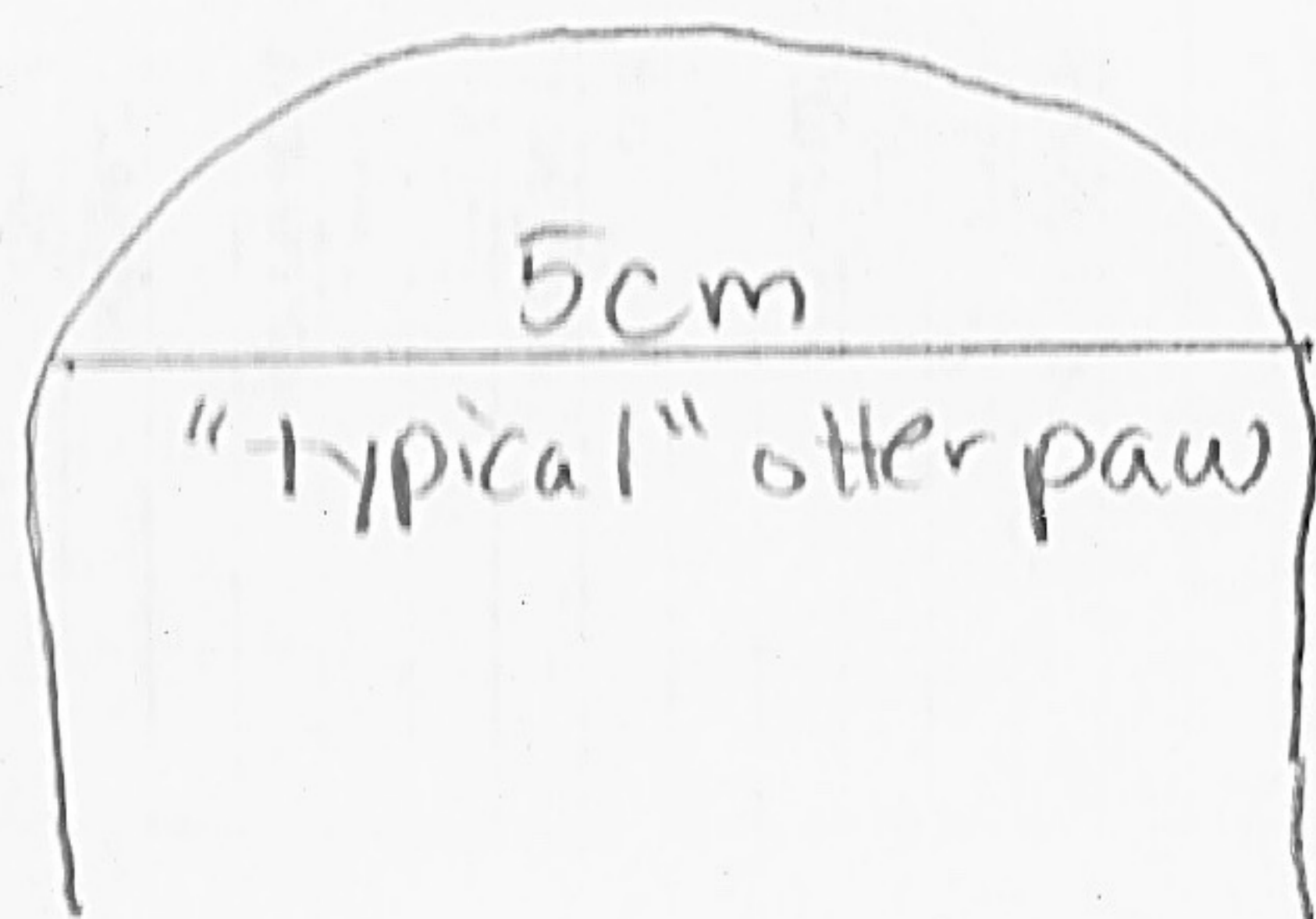

Supplement: Supplementary file 1 [file ECE3-9-3321-s001.pdf]

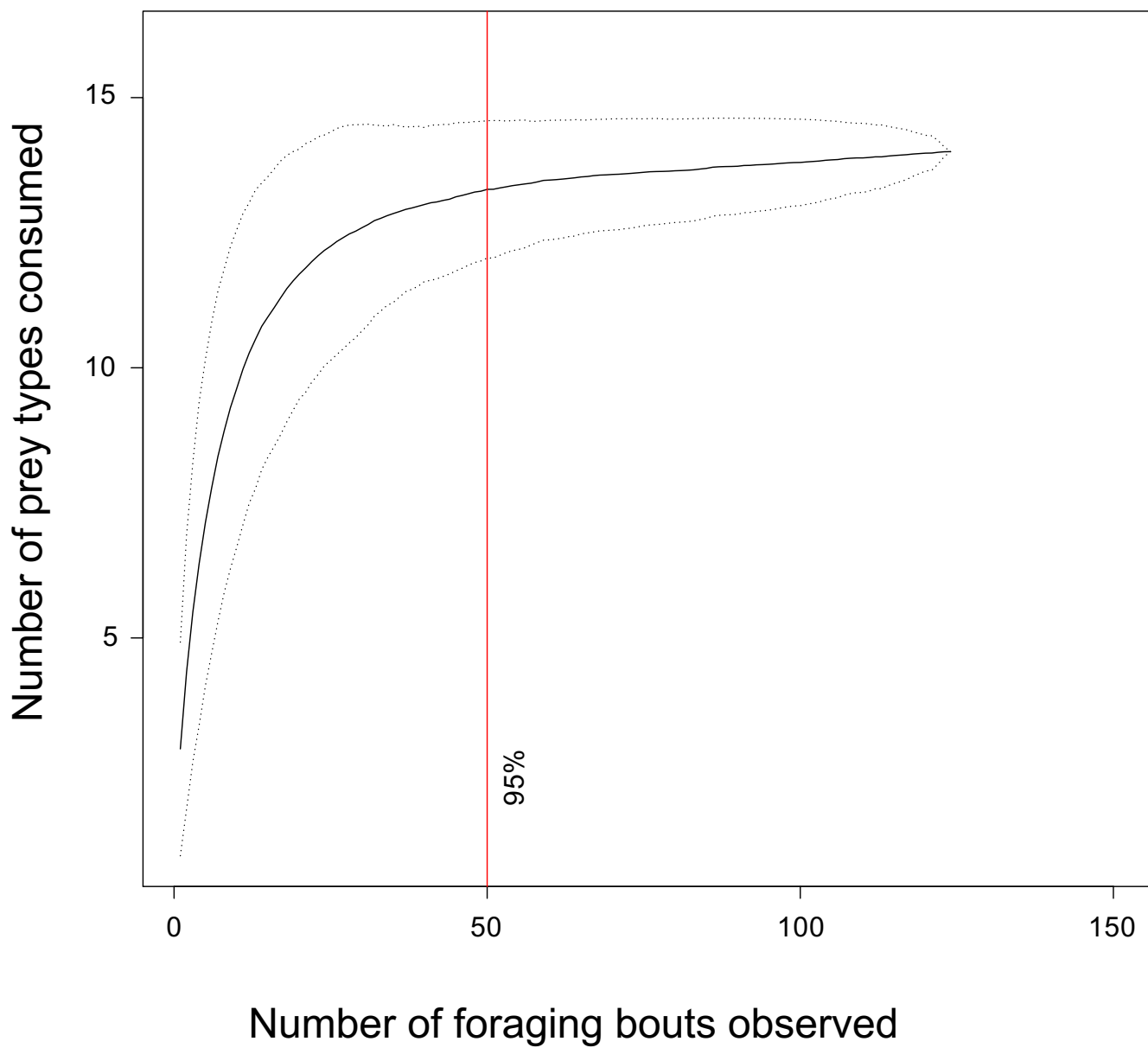

Supplement: Supplementary file 2 [file ECE3-9-3321-s002.pdf]
