# Supplementary material for: Sex and occupation time influence niche space of a recovering keystone predator
Source: Ecol Evol. 2019 Feb 23;9(6):3321–34. doi: 10.1002/ece3.4953 (PMC6434543; doi:10.1002/ece3.4953)
Supplement: Supplementary file 3 [file ECE3-9-3321-s003.docx]

**Table S1.** R-statistics and p-values from pair-wise comparisons of occupation areas assessed by ANOSIM.

|  | **Gosling:**  **27-30 YO** | **McMullins:**  **18-21 YO** | **Simonds:**  **5-8 YO** | **Breadners:**  **3-6 YO** | **Calvert Established:**  **1 YO** |
| --- | --- | --- | --- | --- | --- |
| **McMullins:**  **18-21 YO** | R=0.429; P=0.024* | X | X | X | X |
| **Simonds:**  **5-8 YO** | R=0.958; P=0.029* | R=0.988; P=0.005* | X | X | X |
| **Breadners:**  **3-6 YO** | R=0.354; P=0.057 | R=0.702; P=0.010* | R=0.875; P=0.029* | X | X |
| **Calvert**  **Established:**  **2-4 YO** | R=0.896; P=0.029* | R= 0.980; P=0.005* | R=0.323; P=0.143 | R=0.875; P=0.029* | X |
| **Calvert Initial:**  **1 YO** | R=0.760; P=0.029* | R=0.948; P=0.005* | R=0.938; P=0.029* | R=0.740; P=0.029* | R=0.917; P=0.029* |

*denotes significant different between groups based on *a* = 0.05
