# Supplementary material for: Sex and occupation time influence niche space of a recovering keystone predator
Source: Ecol Evol. 2019 Feb 23;9(6):3321–34. doi: 10.1002/ece3.4953 (PMC6434543; doi:10.1002/ece3.4953)
Supplement: Supplementary file 4 [file ECE3-9-3321-s004.docx]

**Table S2.** Diet, habitat, and depth groups contributing to > 8% within group similarity (proportion by frequency of occurrence) at each occupation area, as identified by SIMPER analysis.

| **Area** | **Average similarity** | **Species** | **Contribution** |
| --- | --- | --- | --- |
| **Gosling:**  **27-30 YO** | 65.35 | mus_sm | 8.23 |
| **McMullins:**  **18-21 YO** | 70.76 | clam_sm | 18.51 |
|  |  | clam_lrg | 14.91 |
|  |  | shallow | 8.71 |
|  |  | crab_sm | 8.22 |
| **Simonds:**  **5-8 YO** | 71.64 | geo_med | 19.00 |
|  |  | geo_sm | 9.60 |
|  |  | clam_lrg | 9.39 |
|  |  | open | 8.16 |
|  |  | urc_lrg | 8.09 |
| **Breadners:**  **3-6 YO** | 72.53 | urc_lrg | 11.89 |
|  |  | urc_sm | 10.26 |
| **Calvert**  **Established:**  **2-4 YO** | 72.29 | clam_lrg | 14.58 |
|  |  | geo_med | 13.06 |
|  |  | urc_lrg | 10.92 |
|  |  | open | 8.10 |
| **Calvert Initial:**  **1 YO** | 69.25 | urc_lrg | 27.41 |
|  |  | clam_sm | 10.65 |
|  |  | urc_sm | 9.05 |
|  |  | open | 8.52 |
