# Supplementary material for: Sex and occupation time influence niche space of a recovering keystone predator
Source: Ecol Evol. 2019 Feb 23;9(6):3321–34. doi: 10.1002/ece3.4953 (PMC6434543; doi:10.1002/ece3.4953)
Supplement: Supplementary file 5 [file ECE3-9-3321-s005.docx]

**Table S3.** Pearson’s correlations of nMDS vectors for occupation areas. Multiple correlation coefficients ≥0.50 bolded.

|  | **Intertidal** | **Shallow** | **Mid** | **Deep** | **Open** | **Kelp** | **Seagrass** | **Yrs occupied** | **Diet diversity** | **Prey size** | **Energy intake** |
| --- | --- | --- | --- | --- | --- | --- | --- | --- | --- | --- | --- |
| **nMDS1** | -0.652 | -0.776 | 0.879 | 0.847 | 0.559 | -0.526 | -0.331 | -0.746 | -0.350 | 0.89 | 0.40 |
| **nMDS2** | 0.052 | 0.197 | -0.180 | 0.243 | -0.035 | -0.211 | 0.333 | 0.274 | 0.139 | -0.078 | -0.48 |
| **Multiple** | **0.655** | **0.800** | **0.897** | **0.881** | **0.561** | **0.567** | 0.469 | **0.795** | 0.377 | **0.895** | **0.625** |
