# Supplementary material for: Sex and occupation time influence niche space of a recovering keystone predator
Source: Ecol Evol. 2019 Feb 23;9(6):3321–34. doi: 10.1002/ece3.4953 (PMC6434543; doi:10.1002/ece3.4953)
Supplement: Supplementary file 6 [file ECE3-9-3321-s006.docx]

**Table S4.** R-statistics and p-values from pair-wise comparisons of sexes assessed by ANOSIM.

|  | **Female** | **Territorial Male** |
| --- | --- | --- |
| **Female** | X | R=0.036  P=0.254 |
| **Bachelor Male** | R=0.602  P=0.001* | R=0.419  P=0.001* |

*denotes significant different between groups based on *a* = 0.05
