# Supplementary material for: Sex and occupation time influence niche space of a recovering keystone predator
Source: Ecol Evol. 2019 Feb 23;9(6):3321–34. doi: 10.1002/ece3.4953 (PMC6434543; doi:10.1002/ece3.4953)
Supplement: Supplementary file 7 [file ECE3-9-3321-s007.docx]

**Table S5.** Diet, habitat, and depth groups contributing to > 8% within group similarity (proportion by frequency of occurrence) of each sex class, as identified by SIMPER analysis.

| **Sex** | **Average similarity** | **Species** | **Contribution** |
| --- | --- | --- | --- |
| **Female** | **60.05** | clam_sm | 11.18 |
|  |  | clam_lrg | 9.83 |
|  |  | shallow | 8.67 |
|  |  | crab_sm | 8.05 |
| **Territorial Male** | **48.23** | clam_sm | 15.31 |
|  |  | shallow | 12.73 |
|  |  | clam_lrg | 10.76 |
|  |  | open | 9.95 |
| **Bachelor Male** | **59.12** | urc_lrg | 14.99 |
|  |  | clam_lrg | 10.84 |
|  |  | geo_med | 10.82 |
|  |  | open | 9.45 |
|  |  | clam_sm | 9.42 |
|  |  | urc_sm | 8.09 |
