# Supplementary material for: Sex and occupation time influence niche space of a recovering keystone predator
Source: Ecol Evol. 2019 Feb 23;9(6):3321–34. doi: 10.1002/ece3.4953 (PMC6434543; doi:10.1002/ece3.4953)
Supplement: Supplementary file 8 [file ECE3-9-3321-s008.docx]

**Table S6.** Pearson’s correlations of nMDS vectors for different sexes. Multiple correlation coefficients ≥0.50 bolded.

|  | **Intertidal** | **Shallow** | **Mid** | **Deep** | **Open** | **Kelp** | **Seagrass** | **Diet diversity** | **Prey size** | **Energy intake** |
| --- | --- | --- | --- | --- | --- | --- | --- | --- | --- | --- |
| **nMDS1** | 0.494 | 0.667 | -0.867 | -0.793 | -0.633 | 0.526 | 0.375 | 0.749 | -0.898 | -0.351 |
| **nMDS2** | 0.253 | -0.033 | 0.189 | -0.155 | 0.086 | 0.191 | -0.377 | -0.027 | 0.096 | 0.320 |
| **Multiple** | **0.555** | **0.667** | **0.887** | **0.808** | **0.639** | **0.560** | **0.532** | **0.749** | **0.903** | **0.475** |
